# 

**Published:** 1833-03

**Authors:** 


### MONTHLY LIST OF MEDICAL BOOKS.

[*Medical Works cannot be entered on this List unless a copy be sent for the purpose, the titles of Books having frequently been sent to us as published, which have not appeared for week, or even months, after.*]

The Cyclopædia of Practical Medicine. Edited by John Forbes, M.D., F.R.S., &c. Alex. Tweedie, M.D., &c., and John Conolly, M.D., &c. Part XIV. (published in Monthly Parts,) February 1833. Containing *Jaundice,* by Dr. Burder; *Kidneys, Diseases of,* Dr. Carter; *Lactation,* Dr. Locock; *Laryngitis,* Dr. Cheyne; *Lepra,* Dr. Houghton; *Leucorrhœa,* Dr. Locock; *Lichen,* Dr. Houghton; *Liver, Inflammation of,* Dr. Stokes; *Malaria, and Miasm,* Dr. Brown; *Malformation of the Heart,* Dr. Williams; *Medicine, Practical Principles of,* Dr. Conolly; *Melæna,* Dr. Goldie; *Melanosis,* Dr. Carswell; *Menorrhagia,* Dr. Locock; *Menstruation,* Dr. Locock.—Large 8vo. Sherwood, Gilbert, and Piper; and Baldwin and Cradock, London.

Outlines of Human Physiology. By HERBERT MAYO, F.R.S., F.G.S., Professor of Anatomy in King's College, London; Surgeon to the Middlesex Hospital, &c. Third Edition.—8vo. pp. 476; numerous engravings. Burgess and Hill, London.

Part II., February 1833. Principles and Illustrations of Morbid Anatomy; adapted to the Elements of M. Andral, and to the Cyclopædia of Practical Medicine (with which it will correspond in size). Being a complete Series of coloured Lithographic Drawings, from Originals by the Author; with Descriptions and summ ary Allusions to Cases, Symptoms, Treatment, &c Designed to constitute an Appendix to Works on the Practice of Physic, and to facilitate the Study of Morbid Anatomy in connexion with Symptoms. By J. HOPE, M.D., F.R.S., Physician to the St. Marylebone Infirmary.—Whittaker and Co., London.

Elements of Surgery, by ROBERT LISTON, Fellow of the Royal Colleges of Surgeons in London and Edinburgh, &c. Part III.—8vo. pp. 409. Longman and Co., London.

Parts XIV. XV. and XVI. The Principles and Practice of Obstetric Medicine, in a Series of Systematic Dissertations on Midwifery and on the Diseases of Women and Children. Illustrated by numerous Plates. By D. D. DAVIS, M.D., M.R.S.L., &c.—4to. John Taylor, London.

The Royal Westminster Ophthalmic Hospital, Chandos street, Charing Cross. Report of the Treasurer for the year 1832; with the Surgeon's Report for the half-year ending 10th January, 1833, drawn up by Messrs. HANCOCK and BEDFORD, House Surgeons.

New Views of the Process of Defecation, and their Application to the Pathology and Treatment of Diseases of the Stomach, Bowels, and other Organs; together with an Analytical Correction of Sir C. BELL's Views respecting the Nerves of the Face. By JAMES O'BEIRNE, M.D., Surgeon Extraordinary to the King, &c.—8vo. pp. 286.—Hodges, Dublin; Longman and Co., London.

A Demonstration of the Nerves of the Human Body. Parts II. and III.—Part II. The Lumbar and Sacral Portions of the Sympathetic and the Nerves of the Abdominal Viscera. Part III. The Cerebral Nerves. By JOSEPH SWAN.—Large folio. Longman, London.

Principles of Physiological Medicine, in the form of Propositions; embracing Physiology, Pathology, and Therapeutics, with Commentaries on those relating to Pathology. By F. J. V. BROUSSAIS, M.D. Translated from the French, by ISAAC HAYS, M.D., and R. E. GRIFFITHS, M.D. &c.—8vo. pp. 594. Carey and Lea, Philadelphia.

Researches on the Pathology and Treatment of some of the most important Diseases of Women. By ROBERT LEE, M.D., F.R.S., &c.—8vo. pp. 220. Highley, London.

The Hunterian Oration; delivered in the Theatre of the Royal College of Surgeons in London, on 14th February, 1833, by JOHN HOWSHIP, Teacher of Surgery, and Surgeon to St. George's Infirmary, &c.

An Explanation of the Causes why Vaccination has sometimes failed to prevent Smallpox, and also a Description of a Method, confirmed by experience, of obviating such Causes. By EDWARD LEESE, Member of the Royal College of Surgeons, and stationary Vaccinator in the National Vaccine Establishment, &c. Part II.—8vo. pp. 36.

---

### APOTHECARIES' HALL.

NAMES OF GENTLEMEN to each of whom the Court of Examiners have granted Certificates:

JAN. 31.

John Hawthornthwaite, of Newcastle-on-Tyne
James Kelly, Ballyshannon, Ireland
George Elisha Newth.

FEB. 7.

Thomas Banks, of Brierley, Staffordshire
Wm. Derington, Hinckley, Leicestershire
John Fountain Elwin, Bath
Brook Fishley, Ilfracombe
Thomas Maw Grisdale, Haxey, Lincolnshire
James John Greer, Bermondsey
Joseph Hayes, Margate
Donald M'Donald, Bristol
Henry Plank
Edward Webb.

FEB. 14.

George Chater, of Norwich
Henry Charles Sutton Dalzell, Weymouth st.
Charles Doble, St. Agnes, Cornwall
Septimus Godson, Hook Norton
Robert Gee, Brackley
Thomas Robinson, Haverill.

FEB. 21.

Roger Turner, of Oldland, W. Lewes
Alexander Farquhar, Chelsea
James Clarke, Pimlico
Thomas Relph Simonds, Wigton
Frederick Shallis Saner, Finsbury square.

### TO CORRESPONDENTS.

*We recommend the perusal of* Dr. *and* Mr. D. Griffin's *excellent series of papers* "On Functional Disorders of the Spinal Cord," *contained in the 65th and 66th volumes of this Journal, to a* "Physician" *at Manchester.*

*We are obliged to* Mr. L—d, *of Paris, for his offer, and we shall be happy to accept any articles he may* volunteer.

To G. G. *The review is well, but rather too severely written. We cannot insert it without he states his name, and gives us an opportunity of judging for ourselves of the justice of his critical tartness. He must let us have the work for a day or two: we cannot procure it without sending to Germany.*

"An Apothecary" *reproaches us for not giving* "*every account of all medical societies.*" *We recommend him to attend them more frequently, and we are quite sure he will approve of our judgment in only giving occasional reports of their meetings. True it is that* "*many learned men attend these conversations;*" *but it is also true that their* "*learning*" *is not always apparent in the discussions that take place at medical societies, for they are too often* "*dull to downright yawning.*"

*We decline answering the question of* "Medicus:" *he appears morbidly sensitive. Any reply to our notice of his work that he chooses to forward to us, shall be inserted: we, of course, reserve to ourselves the liberty of putting in a rejoinder, if we deem it necessary.*

Mr. Ballard's *communications have been received. It is always gratifying to us to find that our exertions are appreciated by our readers. We shall be happy to receive further reports of Cases, &c. from* Mr. B.

Dr. Christie's *valuable and elaborate* Essay on Cholera *has been received; a part of it will be published in our next number.*

